# The bacteriolytic activity of native and covalently immobilized lysozyme against Gram‐positive and Gram‐negative bacteria is differentially affected by charged amino acids and glycine

**DOI:** 10.1002/2211-5463.12591

**Published:** 2019-01-28

**Authors:** Pavel A. Levashov, Darya A. Matolygina, Ekaterina D. Ovchinnikova, Irina Yu Adamova, Darya A. Gasanova, Sergey A. Smirnov, Vladimir A. Nelyub, Natalya G. Belogurova, Vladimir I. Tishkov, Nikolay L. Eremeev, Andrey V. Levashov

**Affiliations:** ^1^ Faculty of Chemistry M.V. Lomonosov Moscow State University Russia; ^2^ Interindustry Engineering Center for Composite Materials N.E. Bauman Moscow State Technical University Russia; ^3^ National Medical Research Center of Cardiology Institute of Experimental Cardiology Ministry of Healthcare of the Russian Federation Moscow Russia; ^4^ POKARD Ltd Moscow Russia

**Keywords:** bacteriolytic activity, charged amino acids, glycine, immobilized lysozyme, lysozyme, native lysozyme

## Abstract

The emergence of new antibiotic‐resistant bacterial strains means it is increasingly important to find alternatives to traditional antibiotics, such as bacteriolytic enzymes. The bacteriolytic enzyme lysozyme is widely used in medicine as an antimicrobial agent, and covalent immobilization of lysozyme can expand its range of possible applications. However, information on the effect of such immobilized preparations on whole bacterial cells is quite limited. Here, we demonstrate the differential effects of glycine and charged (basic and acidic) amino acids on the enzymatic lysis of Gram‐positive and Gram‐negative bacteria by soluble and immobilized lysozyme. Glycine and basic amino acids (histidine, lysine, and arginine) significantly increase the rate of lysis of Gram‐negative *Escherichia coli* cells in the presence of soluble lysozyme, but they do not substantially affect the rate of enzymatic lysis of Gram‐positive *Micrococcus luteus*. Glutamate and aspartate significantly enhance enzymatic lysis of both *E. coli* and *M. luteus*. When using immobilized lysozyme, the effects of amino acids on the rate of cell lysis are significantly reduced. For immobilized lysozyme, the presence of an external diffusion mode on cell lysis kinetics at bacterial concentrations below 4 × 10^8^ colony‐forming units·mL^−1^ was shown. The broadening of the pH optimum of lysozyme activity after immobilization has been demonstrated for both Gram‐positive and Gram‐negative bacteria. The Michaelis constant (*K*_m_) values of immobilized lysozyme were increased by 1.5‐fold for *E. coli* cell lysis and 4.6‐fold for *M. luteus* cell lysis compared to soluble enzyme. A greater understanding of the effect of amino acids on the activity of native and immobilized lysozyme is important for both the development of new materials for medical purposes and elucidating the interaction of lysozyme with bacterial cells. Of particular interest is our finding that lysozyme activity against Gram‐negative bacteria is enhanced in the presence of glycine and charged amino acids over a wide range of concentrations.

**Abbreviations**
*K*_m_: the Michaelis constant
*V*_max_: the maximum rate of reaction

Recently, in connection with the appearance of new antibiotic‐resistant bacterial strains, the use of bacteriolytic enzymes as an alternative to traditional antibiotics has become increasingly important 1, 2, 3. The bacteriolytic enzyme lysozyme has been known for a long time and is widely used in medicine as an antimicrobial agent. Obtaining and using preparations of covalently immobilized lysozyme is an important task, expanding the range of possible applications of this enzyme. The covalently immobilized enzyme can, for example, potentially be used for patient's blood plasma and whole blood purification by extracorporeal therapy procedures. Currently, there are some examples of covalent immobilization of lysozyme 4, 5, 6, but information about the effect of such preparations on whole bacterial cells is quite limited. In our recent work, we showed that soluble lysozyme can significantly change its activity against *Escherichia coli* in the presence of glycine and such charged amino acids as lysine, arginine, and glutamate 7. Understanding the peculiarities of the effect of different effectors, contained in living organisms, on enzyme activity is extremely important both for the development of new drugs and for the comprehension of the functioning of bacteriolytic enzymes in the immune system. In this paper, we set ourselves the task of expanding the list of the charged amino acids under study and also comparing their effects on the lysis rate of bacteria under the action of native (soluble) and immobilized lysozyme. For an adequate understanding of the characteristics of lysozyme action in real conditions, it is important to conduct research on whole bacterial cells, and not on artificial substrates. As model substrates, we used the Gram‐positive bacterium *Micrococcus luteus* and the Gram‐negative bacterium *E. coli*. As a polymer matrix for immobilization of lysozyme, we used agarose WB200 with a granule diameter of 200 μm, for which its hemocompatibility and suitability for use in medicine in extracorporeal blood purification procedures was previously shown 8.

## Materials and methods

### Materials and equipment

The following reagents were used: lysozyme from chicken eggs, *M. luteus* (lyophilized cells), NaN_3_, MES, NaIO_4_, 1,6‐diaminohexane, sodium acetate, Tris, NaBH_4_ (Sigma‐Aldrich, St Louis, MO, USA); KH_2_PO_4_, K_2_HPO_4_, HCl, yeast extract, l‐arginine (Helicon, Moscow, Russia); agar (Ferak, Berlin, Germany); Workbeads 200SEC polymer matrix (Bio‐Works, Uppsala, Sweden); glutaraldehyde, NaOH, NaCl, (Panreac, Castellar del Vallès, Spain); glycine (Fluka, Munich, Germany); l‐histidine, l‐lysine (Serva, Heidelberg, Germany); NaHCO_3_, acetic acid, l‐aspartic acid (Reahim, Moscow, Russia), and sodium l‐glutamate (HongMei, Shenyang, China). Museum strain *E. coli* JM109 was provided by J. Messing (Waksman Institute, Piscataway, NJ, USA). All solutions were prepared in bidistilled water. The following equipment was used in the studies: a UV‐1800 spectrophotometer (Shimadzu, Kyoto, Japan), a TV‐80‐1 air circulation oven with thermostat control (MedLife, Kasimov, Russia), an LT‐105a water bath with thermostat control (LOIP, Saint‐Petersburg, Russia), an OH‐PA64 analytical balance (Ohaus, Parsippany‐Troy, NJ, USA), a Multi Bio RS‐24 rotator shaker (BioSan, Riga, Latvia), and a MiniSpin centrifuge (Eppendorf, Berzdorf‐Wesseling, Germany).

### Amination of agarose matrix

The standard method was taken as the base 9. The matrix was washed with water and a two‐fold volume (relative to the volume of the matrix) of 2% NaIO_4_ solution was added. The mixture was incubated at 20 °C for 2 h on a rotary shaker (5 r.p.m.) and the matrix was washed with a 20‐fold volume of distilled water. A single volume of 2 m 1,6‐diaminohexane solution was added to the activated matrix followed by incubation of the mixture at 20 °C for 2 h on a rotary shaker (5 r.p.m.). A double volume of freshly prepared 0.5% NaBH_4_ aqueous solution was added to the obtained preparation and incubated for 30 min while stirring, then another similar portion of freshly prepared NaBH_4_ solution was added and the mixture incubated for an additional 30 min. Next, the preparation was washed with a five‐fold volume of 1 m NaCl solution and a 10‐fold volume of a KH_2_PO_4_–K_2_HPO_4_ buffer (10 mm, pH 7.0, 130 mm NaCl).

### Immobilization of lysozyme on the aminated matrix

For the attachment of lysozyme to the insoluble polymeric matrix, we used the amino groups not involved in catalysis of lysine residues (three such residues are exposed on the surface of a protein globule 10). To 10 mL of 50% aminated matrix suspension in a NaHCO_3_–NaOH buffer (30 mm, pH 10.0), 0.56 mL of 25% glutaraldehyde solution was added and stirred at 25 °C for 30 min on a rotary shaker (5 r.p.m.). Then the gel was washed in a glass filter with 50 mL of a NaHCO_3_–NaOH buffer (30 mm, pH 10.0), transferred to a separate container and 10 mL of a lysozyme solution (7.5 mg·mL^−1^) added to the same buffer. The mixture was incubated at 25 °C for 3 h on a rotary shaker (5 r.p.m.). The resulting sorbent was twice treated with 10 mL of a 0.5% NaBH_4_ solution – the time of each incubation being 20 min. After each blocking, the sorbent was washed with 200 mL of distilled water. Finally, the preparation was washed with 150 mL of KH_2_PO_4_–K_2_HPO_4_ buffer (10 mm, pH 7.0, 130 mm NaCl).

### Determination of immobilized lysozyme carrier capacity

Carrier capacity was determined by the difference between the added amount of lysozyme and its amount in the supernatant after the immobilization procedure. The concentration of lysozyme was determined spectrophotometrically by the optical absorption of the solution at 280 nm.

### Immobilized lysozyme storage

The preparation was stored at 5 °C as a 50% suspension (containing 50% sediment by volume) in KH_2_PO_4_–K_2_HPO_4_ buffer (10 mm, pH 7.0, 130 mm NaCl). Before the experiments, the preparation was washed in a glass filter with a 10‐fold volume of the same buffer. After the preparation had been stored for more than 2 weeks, 0.3% w/w of NaN_3_ was added to the mixture. The activity of the immobilized lysozyme did not change in the range of experimental error during 3 months of storage.

### Substrate preparation

The growth of *E. coli* cells was carried out in accordance with the standard method 11. A cell suspension (~ 10^9^ cells·mL^−1^) in 0.15 m NaCl was frozen by immersing the tubes in liquid nitrogen and then stored at −70 °C for no more than 3 weeks. Samples of *E. coli* cells were defrosted immediately prior to the experiment. The suspension of *M. luteus* cells was prepared by adding 5 mg of dried cells in 10 mL of Tris–MES–acetate buffer (0.01 m, pH 8.8) at a temperature of 20 °C. Before use, suspensions of *E. coli* and *M. luteus* cells were centrifuged for 5 min at 1358 ***g*** at 5 °C and resuspended in a Tris–MES–acetate buffer (0.01 m, pH 8.8).

### Determination of soluble lysozyme activity on cells

The bacteriolytic activity of lysozyme was determined by the turbidimetric method as decreasing of the attenuance of the cell suspension at a wavelength of 650 nm (*D*_650_). The rate of change in attenuance is directly proportional to the rate of cell lysis 12, 13. The measurements were carried out in 0.5 mL cuvettes with a ground stopper at 37 °C in a Tris–MES–acetate buffer (0.01 m, pH 8.8). The initial attenuance of bacterial cells in the cuvette was 0.05–0.8; lysozyme concentration was 0.1 μg·mL^−1^. After the enzyme was added to the cuvette, the kinetics of the change in attenuance was recorded for 5–7 min and the initial velocities were determined in the first 2–3 min interval. For the correction of the rate of lysis on the background change in the attenuance, control experiments were performed without the addition of an enzyme. The enzymatic cell lysis rate is proportional to the lysozyme concentration in the range up to 1.0 μg·mL^−1^.

### Determination of the immobilized lysozyme activity on cells

Similarly, as in the case of soluble lysozyme, the decrease in attenuance of the cell suspension at 650 nm was measured. The measurements were carried out in the same buffer mixture and at the same temperature. The initial attenuance of bacterial cells in a cuvette was 0.05–0.8. Immobilized lysozyme preparation was added to the mixture of total volume 10 mL (final concentration 35 μL·mL^−1^). The reaction mixture was incubated in test tubes (10 mL each) in a thermostat at 37 °C on a rotary shaker at 10 r.p.m. (at speeds of more than 14 r.p.m., an effect of cell destruction without the action of the enzyme is apparent, probably due to the mechanical action of the drug pellet on the cells). Samples (1 mL) were taken from the mixture every 2 min, leaving the rest of the mixture for further incubation. Selected samples of 1 mL were placed in tubes, the particles of the immobilized enzyme were allowed to settle (0.5 min), and the attenuance of the supernatant (suspension of cells without immobilized enzyme) was then measured. The dependence of the change in attenuance over time was plotted for 16 min, and the rate of change of attenuance with time was determined from the slope of the dependence. For the correction of the lysis rate on the background attenuance change, control experiments were performed by adding to the mixture a matrix without lysozyme. The enzymatic cell lysis rate is proportional to the amount of the preparation of the immobilized enzyme in the range up to 90 μL·mL^−1^ of the mixture.

### Measurement of native and immobilized lysozyme activity in the presence of effectors

A similar experiment with a native and immobilized lysozyme was performed using amino acids in concentrations from 0.1 to 15 mm in a cuvette. Stock solutions were adjusted to pH 8.8. Control experiments have shown that cell lysis was not observed when adding effectors in the absence of lysozyme. The number of bacterial cells added to the reaction mixture was selected in such a way that the initial attenuance was 0.5–0.55.

### Measurement of native and immobilized lysozyme activity at different pH values

Activities of native and immobilized enzyme were measured, as described above, at different pH values (6.5–9.25). The number of bacterial cells added to the reaction mixture was selected in such a way that the initial attenuance was 0.5–0.55.

## Results and Discussion

In experiments for the determination of lysozyme activity we used 0.1 μg·mL^−1^ of soluble enzyme or 35 μL·mL^−1^ of the preparation of immobilized enzyme – in this case, the rates of cell lysis is comparable. Thirty‐five microlitres of the immobilized enzyme preparation contains ~ 525 μg of protein, which is 5250 times more than in the experiment with soluble lysozyme. Presumably, due to the large size of the substrate particles (bacterial cells), only lysozyme on the surface of the granules is sterically able to show enzymatic activity. Immobilization of excess amounts of lysozyme, in our opinion, does not prevent the technological use of the preparation, since lysozyme is a relatively inexpensive commercially available product. If necessary, further developments can use polymers with low‐porosity granules to attach lysozyme to the surface of the particles only.

**Figure 1:**
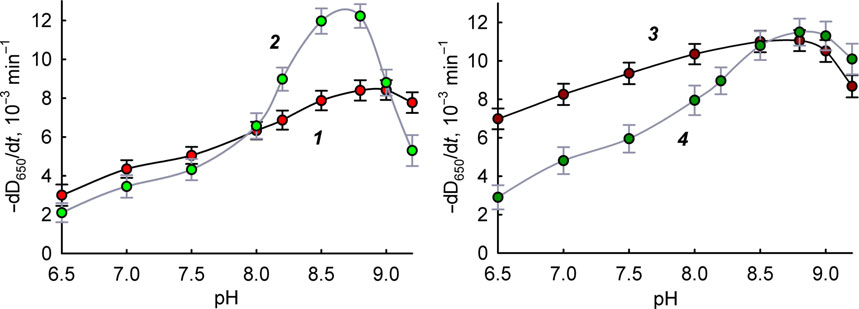
The pH dependence of the lysis rate of bacteria in the presence of lysozyme. Experimental data are presented as mean and 95% confidence interval calculated by Student's *t*‐test, *n* = 5. 1: *M. luteus* in the presence of free lysozyme; 2: *E. coli* in the presence of free lysozyme; 3: *M. luteus* in the presence of immobilized lysozyme; 4: *E. coli* in the presence of immobilized lysozyme.

Figure 1 shows a comparison of pH dependences of the free and immobilized lysozyme activity in the case of different bacterial cells as a substrate. It is apparent that immobilized lysozyme is characterized by smaller changes in activity with a change in pH and broadening of the optimum activity. This phenomenon is especially noticeable for *M. luteus* cells. pH‐dependence smoothing may be associated in our opinion either with distortions of the conformation of the enzyme during immobilization or with the influence of mass transfer kinetics (diffusion) on the enzymatic kinetics. In any case, it is even preferable for medical use to have an extended effective pH range for the action of the immobilized enzyme. Changes in the pH profile of lysozyme activity during immobilization were also noted in studies of its non‐covalent immobilization on cellulose nanoparticles 14 and covalent and non‐covalent immobilization on chitosan 4. It should be noted that even in the case of soluble lysozyme, the interaction of the enzyme with the substrate is a complex process in which the surface charges of the enzyme and the substrate play an important role. pH activity profiles can be complex, depending on the ionic strength and type of substrate 15. In the case of different substrates, the thermodynamic parameters of their binding to the enzyme may depend on pH in different ways 16. In the case of an immobilized enzyme, the nature of the influence of various factors is even more complicated. For example, for the non‐covalent immobilization of lysozyme on silica nanoparticles, it was shown that the structure and properties of lysozyme may vary significantly depending on the shape of the sorbing surface and on the pH 17. The type of pH dependence of immobilized lysozyme activity is probably the cumulative result of various factors. This phenomenon requires further careful study but goes beyond the basic premise of this work.

**Figure 2:**
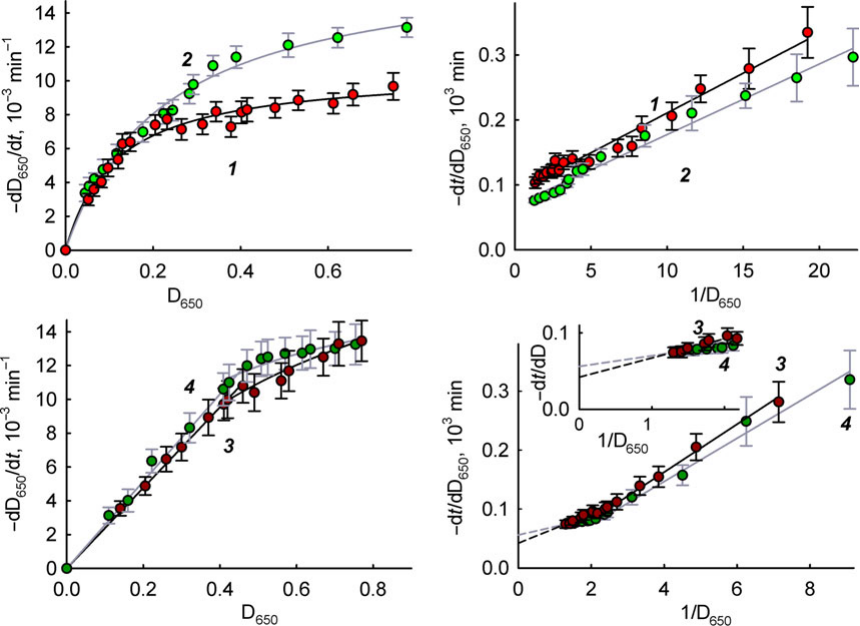
The dependence of the lysis rate of bacteria in the presence of lysozyme on the concentration of bacteria in the direct and inverse coordinates. Experimental data are presented as mean and 95% confidence interval calculated by Student's *t*‐test, *n* = 5. 1: *M. luteus* in the presence of free lysozyme; 2: *E. coli* in the presence of free lysozyme; 3: *M. luteus* in the presence of immobilized lysozyme; 4: *E. coli* in the presence of immobilized lysozyme.

Figure 2 shows the dependence of the lysis rate of cells on their quantity (substrate concentration). For ease of presentation, we left the cell concentration values in units of attenuance of the suspension at 650 nm (*D*_650_). In the range 0–1, the *D*_650_ value is proportional to the cell concentration, adjusted for coefficients for different bacteria 12, 13. As can be seen from Fig. 2, for the soluble enzyme the dependence obeys the Michaelis–Menten equation and the values of the maximum rate of reaction (*V*_max_) and Michaelis constant (*K*_m_) can be determined. The *K*_m_ values are almost the same for *M. luteus* and *E. coli* – 0.14 ± 0.02 and 0.17 ± 0.02 (in attenuance units), respectively. The values of *V*_max_ are also close for *M. luteus* and *E. coli*, respectively (1.2 ± 0.1) × 10^−2^ and (1.5 ± 0.1) × 10^−2^ min^−1^.

For immobilized lysozyme, we see a deviation from Michaelis–Menten hyperbolic dependence in direct coordinates and a break in double reciprocal coordinates (Fig. 2, lower part). The dependences of the cell lysis rate on the substrate concentration in the presence of immobilized lysozyme have a distinct two‐phase character. However, in the range of cell concentration 0.4–0.75 *D*_650_, this dependence obeys the Michaelis–Menten equation, which makes it possible to determine the values of *V*_max_ and *K*_m_. The *V*_max_ values for *M. luteus* and *E. coli* for the immobilized enzyme are (2.5 ± 0.4) × 10^−2^ and (1.8 ± 0.3) × 10^−2^ min^−1^, respectively. As in the case of the soluble enzyme, the *V*_max_ values measured under the same conditions differ a little for different bacteria. However, in the case of free enzyme, the *V*_max_ value for *E. coli* was ~ 25% more compared to the *V*_max_ value for *M. luteus*. On the contrary, for immobilized enzyme, the value of *V*_max_ for *M. luteus* is ~ 40% more than for *E. coli*. Thus, it can be said that with the immobilization of the enzyme, *V*_max_ falls more strongly in the case of the substrate *E. coli*. The *K*_m_ values for the lysis of *M. luteus* and *E. coli* by immobilized lysozyme are 0.65 ± 0.05 and 0.25 ± 0.03 (in attenuance units), respectively. Thus, immobilization of lysozyme leads to increasing *K*_m_ values by 1.5 times in the case of *E. coli* and 4.6 times in the case of *M. luteus*. Such a difference in the change in *K*_m_ values for different substrates is probably due to the difference in cell shape and surface charge distribution, which may potentially cause some interaction difficulties between *M. luteus* cells and immobilized lysozyme.

At low cell concentrations (*D*_650_ ≤ 0.4), in both direct and double reciprocal coordinates, the rate of bacterial lysis by immobilized enzyme is directly proportional to the concentration of cells, which indicates a first order reaction for the substrate. According to the classical two‐stage scheme of enzymatic catalysis, such a case may occur at concentrations of the substrate much smaller than the value of the Michaelis constant ([*S*]_o_ < 0.1 *K*_m_). In our case, the break is observed under the conditions of [*S*]_o_ ≈ *K*_m_ and, in our opinion, is associated with external diffusion difficulties when working with immobilized lysozyme, as for a number of other immobilized enzymes 18, 19, 20. For stationary diffusion from immiscible layer to the plane surface with a constant diffusion coefficient, the solution of Fick's first law leads to the simplest equation for the flow of a diffusing substance on the unit of area:

(1) $$J=-D_{s}\frac{{\left[S\right]}_{o}-{\left[S\right]}_{l}}{l}.$$

where *l* is the thickness of the non‐mixed layer, [*S*]_o_ and [*S*]_l_ are the concentrations of the substrate in the solution and on the surface, and *D*_s_ is the diffusion coefficient of the substrate. The enzymatic reaction on the surface is described by the Michaelis–Menten equation, where the substrate concentration is [*S*]_l_:

(2) $$V=\frac{V_{\max}{\left[S\right]}_{l}}{K_{m}+{\left[S\right]}_{l}}.$$

In the steady state, the rate of diffusion of the substrate and the rate of the enzymatic reaction are equal to each other (*J* = −*V*, since the diffusion process is aimed at increasing the concentration of [*S*]_l_, and the enzymatic reaction at reducing it), thus:

(3) $$D_{s}\frac{{\left[S\right]}_{o}-{\left[S\right]}_{l}}{l}=\frac{V_{\max}{\left[S\right]}_{l}}{K_{m}+{\left[S\right]}_{l}}.$$

If the mass transfer is faster than the catalytic reaction, then the reaction proceeds in the kinetic mode and obeys the Michaelis–Menten equation (observed reaction rate *V*′ = *V*_enz_, Eqn (2)). On the contrary, if a chemical reaction goes much faster than diffusion, then the observed reaction is diffusion controlled, and the reaction rate is of the first order in the substrate (observed reaction rate *V*′ = *V*_diff_, Eqn (1)).

The exact solution of Eqn (3) relative to [*S*]_l_ is as follows (the choice of the root sign is determined by the inequality [*S*]_o_ > [*S*]_l_ > 0):

(4) $${\left[S\right]}_{l}=\frac{{\left[S\right]}_{o}-\frac{V_{\max}}{h_{s}}-K_{m}}{2}+\sqrt{{\left(\frac{{\left[S\right]}_{o}-\frac{V_{\max}}{h_{s}}-K_{m}}{2}\right)}^{2}+K_{m}{\left[S\right]}_{o}},$$

where *h*_s_ = *D*_s_/*l*, or in dimensionless form:

(5) $$\frac{{\left[S\right]}_{l}}{{\left[S\right]}_{o}}=\frac{1-\frac{K_{m}}{{\left[S\right]}_{o}}\left(1+\theta \right)}{2}+\sqrt{{\left(\frac{1-\frac{K_{m}}{{\left[S\right]}_{o}}\left(1+\theta \right)}{2}\right)}^{2}\frac{K_{m}}{{\left[S\right]}_{o}}}.$$

The introduced dimensionless parameter θ = *V*_max_/(*K*_m_ *h*_s_) = *k*_kat_[*E*]_o_ *l*/(*K*_m_ *D*_s_). Analysis of Eqn (5) shows that the diffusion or kinetic mode of the enzymatic reaction determines the values of the parameters θ and *K*_m_/[*S*]_o_.

If θ ≪ 1, then [*S*]_l_/[*S*]_o_ = 1. This means that the concentration of the substrate on the interface is the same as in the solution. In other words, the system operates in the kinetic mode, and diffusion processes do not affect the observed enzymatic reaction.

If θ ≫ 1, there are two possible solutions depending on the value of the parameter *K*_m_/[*S*]_o_. When *K*_m_/[*S*]_o_ ≪ 1 (the concentration of the substrate in the solution is much greater than the value of *K*_m_) and at the same time (*K*_m_/[*S*]_o_)θ ≪ 1, it follows from Eqn (5) that [*S*]_l_/[*S*]_o_ → 1 (the concentration of the substrate on the surface of the particle is approximately equal to its concentration in the solution) and the system operates in the kinetic mode. When *K*_m_/[*S*]_o_ ≥ 1 (the concentration of the substrate in the solution is comparable to or less than *K*_m_), we have [*S*]_l_/[*S*]_o_ ≪ 1, that is, the concentration of the substrate on the surface of the particle is much less than its concentration in the solution and the system works in diffusion mode. Thus, the transition from the diffusion to the kinetic regime should occur at concentrations of the substrate comparable to the value of *K*_m_. As one can see from Fig. 2, the transition to the diffusion regime is observed at *D*_650_ values < 0.4 (cell concentrations ≈ 4 × 10^8^ colony‐forming units·mL^−1^ 21) when *K*_m_/[*S*]_o_ becomes ≥ 1.

**Figure 3:**
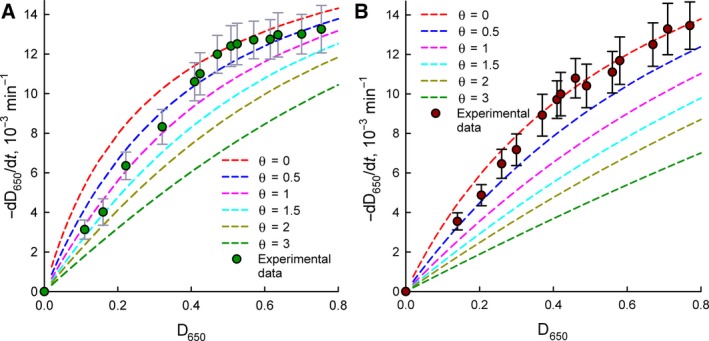
Comparison of experimental (circles) and calculated (dash lines) data for the dependence of bacterial lysis rate in the presence of immobilized lysozyme on the substrate concentration. Calculations were done in accordance with Eqn (5) at different values of parameter θ. (A) *Escherichia coli*; (B) *Micrococcus luteus*. Experimental data are presented as mean and 95% confidence interval calculated by Student's *t*‐test, *n* = 5.

Figure 3 presents a comparison of the experimental activity data for the immobilized enzyme and the theoretical curves obtained using the Michaelis–Menten equation with the recalculation of the substrate concentration (*D*_650_) according to Eqn ( (5)) for different θ values. The theoretical dependences confirm the tendencies of changes in the kinetics under diffusional constraints; however, complete correlation between the experimental and calculated data is not observed. In the case of [*S*] > *K*_m_, the experimental data are in agreement with the theoretical curve for θ = 0. In the case of [*S*] < *K*_m_, the experimental data are in good agreement with the theoretical curve at θ = 1.5 for *E. coli* and θ = 0.5 for *M. luteus*. The simple Fick equation probably does not take into account all aspects of the real kinetics of mass transfer of the substrate in this case. A more detailed explanation of the features of the kinetics of the diffusion regime in this system is beyond the scope of this work.

**Figure 4:**
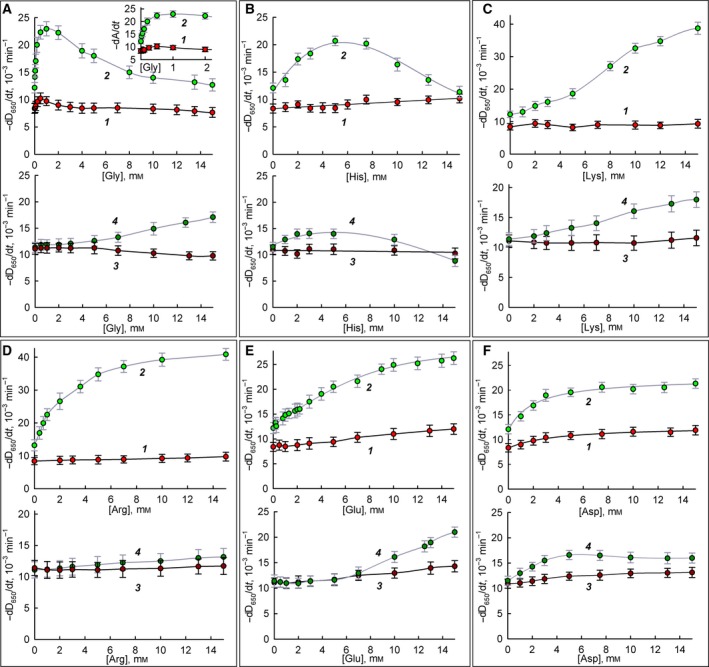
The dependence of bacteria lysis rate in the presence of lysozyme on the concentration of amino acids. (A) Glycine; (B) histidine; (C) lysine; (D) arginine; (E) glutamate; and (F) aspartate. Experimental data are presented as mean and 95% confidence interval calculated by Student's *t*‐test, *n* = 5. 1: *M. luteus* in the presence of free lysozyme; 2: *E. coli* in the presence of free lysozyme; 3: *M. luteus* in the presence of immobilized lysozyme; 4: *E. coli* in the presence of immobilized lysozyme.

Figure 4A,B presents data on the effect of glycine and histidine on the lysis rate of bacterial cells in the presence of lysozyme preparations. Glycine and histidine do not affect the rate of *M. luteus* lysis in the case of both free and immobilized enzyme. The rate of enzymatic lysis of *E. coli* cells by free lysozyme is significantly enhanced in the presence of these effectors. The observed dependences have a bell shape, with a maximum lysis rate increase of almost double at concentrations of 0.5 mm glycine and 5 mm histidine. In the case of immobilized lysozyme, increased lysis of *E. coli* in the presence of these amino acids is also observed, but the effect is less pronounced. For example, to increase the lysis rate 1.5 times, only 0.1 mm glycine in the case of the soluble enzyme and 15 mm glycine in the case of an immobilized enzyme (150 times higher concentration) is necessary. Under the action of histidine, the greatest increase in the rate of *E. coli* cell lysis is achieved at the same effector concentration (5 mm) with both the soluble and immobilized enzymes; however, for immobilized enzymes, the maximum effect of increased cell lysis is only ~ 25% compared to ~ 75% for soluble lysozyme. The molecular reasons for such effects of glycine and histidine are unknown. Regarding glycine, there is information that it alone can inhibit the growth of some bacteria 22. Histidine residues can be part of antibacterial cationic peptides, although replacing arginine on histidine in a peptide usually limits the range of conditions for antibacterial action 23. Thus, note that both glycine and histidine have a notable effect on the enzymatic lysis of Gram‐negative bacteria, without having a significant effect in the case of Gram‐positive ones. It can be assumed that these effectors somehow facilitate the penetration of lysozyme through the outer membrane into the periplasm of Gram‐negative bacteria. These effects require further close study since the mechanism of lysozyme penetration to peptidoglycan is currently not fully understood 24. Also, note that all effects that are explicitly expressed with soluble lysozyme are reduced in the case of immobilized lysozyme.

Figure 4C,D presents data on the effect of the basic amino acids lysine and arginine on the lysis rate of various bacteria in the presence of soluble and immobilized lysozyme. We see that in the case of soluble lysozyme, these two effectors have a significant effect on the lysis rate of Gram‐negative *E. coli* bacterial cells similar to what was observed for glycine and histidine. For immobilized enzyme, the effect of lysine on the lysis rate of *E. coli* decreases, and the effect of arginine disappears altogether. At the same time, the rate of lysis of Gram‐positive *M. luteus* in the presence of both soluble and immobilized lysozyme is practically independent of the presence of lysine and arginine. It is known that lysine and arginine are often present in antibacterial peptides 25, 26. It can be assumed that these free amino acids can improve the binding of lysozyme to the negatively charged surface of the bacterium, enhancing lysis. It is also possible that these effectors facilitate the penetration of lysozyme into the periplasm of the cell.

Figure 4E,F presents data on the effect of the acidic amino acids glutamate and aspartate on the lysis rate of the bacteria in the presence of soluble and immobilized lysozyme. In this case, we see a significant effect on the rate of cell lysis both Gram‐negative *E. coli* and Gram‐positive *M. luteus* for soluble lysozyme. For immobilized enzyme, these effects are reduced but nevertheless are also observed. As we see, there is no such dramatic difference in the effect of glutamate and aspartate on the lysis of Gram‐negative *E. coli* and Gram‐positive *M. luteus* cells, unlike other amino acids used. One can assume that the mechanism of action of glutamate and aspartate, which have additional negatively charged carboxyl groups, is fundamentally different from the mechanism of action of glycine and basic amino acids.

The present study demonstrated previously unknown effects of free amino acids on the antibacterial activity of lysozyme, which undoubtedly should be further studied in depth since a new, previously unknown side of the possibility of regulating the action of the body's antibacterial enzymes opens up. Also, knowledge of enhancing the action of lysozyme in the presence of studied effectors can help in the development of new highly effective antibacterial pharmaceutical preparations. In this work, it was also shown that immobilization of lysozyme leads to the broadening of the pH optimum of enzyme activity and reduction of the effects of potential activators on the enzyme. This information should also be taken into account when developing antibacterial composite materials based on lysozyme when predicting the effectiveness of immobilized lysozyme in various conditions.

## Author contribution

PAL, VAT, IYA and AVL conceived and designed the project. PAL, DAM, EDO, DAG, SAS and NGB acquired the data. PAL, VIT, IYA, VAN, NLE and AVL analyzed and interpreted the data. PAL, VAT and NLE wrote the paper. All the authors have read and approved the manuscript.

## Conflict of interest

The authors declare no conflict of interest.
